# The role of TXNIP in cancer: a fine balance between redox, metabolic, and immunological tumor control

**DOI:** 10.1038/s41416-023-02442-4

**Published:** 2023-10-04

**Authors:** Jinhai Deng, Teng Pan, Zaoqu Liu, Caitlin McCarthy, Jose M. Vicencio, Lulu Cao, Giovanna Alfano, Ali Abdulnabi Suwaidan, Mingzhu Yin, Richard Beatson, Tony Ng

**Affiliations:** 1 Richard Dimbleby Laboratory of Cancer Research, School of Cancer & Pharmaceutical Sciences, King’s College London, London, UK; 2 Clinical Research Center (CRC), Clinical Pathology Center (CPC), Chongqing University Three Gorges Hospital, Chongqing University, Wanzhou, Chongqing, China; 3 Longgang District Maternity & Child Healthcare Hospital of Shenzhen City (Longgang Maternity and Child Institute of Shantou University Medical College), Shenzhen, 518172 China; 4 Department of Interventional Radiology, The First Affiliated Hospital of Zhengzhou University, Zhengzhou, China; 5 Department of Rheumatology and Immunology, Peking University People’s Hospital and Beijing Key Laboratory for Rheumatism Mechanism and Immune Diagnosis (BZ0135), Beijing, China; 6 Centre for Inflammation and Tissue Repair, UCL Respiratory, Division of Medicine, University College London (UCL), Rayne 9 Building, London, WC1E 6JF UK; 7 UCL Cancer Institute, University College London, London, UK; 8 Cancer Research UK City of London Centre, London, UK

**Keywords:** Cancer metabolism, Cancer microenvironment

## Abstract

Thioredoxin-interacting protein (TXNIP) is commonly considered a master regulator of cellular oxidation, regulating the expression and function of Thioredoxin (Trx). Recent work has identified that TXNIP has a far wider range of additional roles: from regulating glucose and lipid metabolism, to cell cycle arrest and inflammation. Its expression is increased by stressors commonly found in neoplastic cells and the wider tumor microenvironment (TME), and, as such, TXNIP has been extensively studied in cancers. In this review, we evaluate the current literature regarding the regulation and the function of TXNIP, highlighting its emerging role in modulating signaling between different cell types within the TME. We then assess current and future translational opportunities and the associated challenges in this area. An improved understanding of the functions and mechanisms of TXNIP in cancers may enhance its suitability as a therapeutic target.

## Introduction

Reduction-oxidation (redox) reactions, where the exchange of electrons from one compound to another occurs within the same reaction, are critical to cellular life. They are required for multiple biological processes from metabolism to enzymic function. These potent reactions carry risk, with dysregulated redox management being shown to be involved in the initiation and progression of multiple pathologies, including metabolic, neurodegenerative, cardiovascular, and neoplastic diseases [1–3]. Thioredoxins (TXN and TXN2), together with glutathione, constitute the major thiol antioxidants that ensure these reactions take place with limited local damage [4]. Thioredoxin-interacting protein (TXNIP) has been shown to bind and inhibit thioredoxins (Trx) [5–7]. Consequently, dysregulation of this TXNIP-Trx axis is strongly associated with metabolic diseases [8–10].

In addition to being a major redox regulator, TXNIP has also been identified as a tumor suppressor gene (TSG), and its expression is reduced in a wide range of primary tumors and cancer cell lines compared to normal tissue and cell lines, respectively [11–16]. Its function as a TSG is also supported by the observation that *Txnip*-deficient mice have a 40% higher incidence of spontaneously developing hepatocellular carcinoma (HCC) [17]. However, different studies in a variety of different cancers, utilising a variety of different techniques have reached different conclusions, suggesting that the role of TXNIP is complex in cancer and may have different implications depending on cancer type and stage of disease (Table 1). Moreover, single-cell RNA sequencing of T-cell lymphoma reveals that low levels of TXNIP expression correlate with malignancy and disease progression [18]. Indeed, accumulating evidence suggests that downregulation of TXNIP is associated with poorer prognosis in diffuse large B-cell lymphoma [19].

**Table 1:** Summary of complicated roles of TXNIP in cancer types.

Pro-/antitumor	Cancer Type	Primary results	Conclusion
Antitumor	Adenoid Cystic Carcinoma (ACC)	Downregulated in ACC	*TXNIP* has a frameshift insertion in its arrestin domain (L129fs) [189].
Antitumor	Acute Myeloid Leukemia (AML)	Silenced by epigenetic regulators	TXNIP mediates histone methyltransferase inhibitor-induced apoptosis by regulating ROS [84] and induced cell cycle arrest [190], apoptosis [191] and drug resistance [192].
Antitumor	Bladder cancer	Decreased in human bladder cancers and in the N -butyl- N –(4-hydroxybutyl) nitrosamine (BBN) -induced mice bladder cancer model	Knock-out TXNIP facilitates CXCR4- induced ERK phosphorylation, promoting bladder carcinogenesis [11]; induced by D-allose to inhibit bladder cancer cell viability [193].
Antitumor	Glioma	Lower expression in high-grade compared to low-grade gliomas (LGG).	High TXNIP expression is associated with favorable clinical outcome in pediatric LGGs [194] Overexpressing TXNIP sensitises tumors to chemotherapy treatment [114].
Antitumor	Breast cancer	Repressed by estrogen and in triple negative breast carcinoma (TNBC) doxorubicin-resistant tissues and cells	TXNIP expression reprogrammes the metabolic phenotype of estrogen receptor (ER) positive breast cancers [54]. TXNIP overexpression in TNBC cells causes ROS-mediated DNA damage and reverses chemotherapy resistance [195].
Antitumor	Cervical cancer	Repressed by DNA methylation regulator	TXNIP expression is decreased due to DNA methylation [196], facilitating the tumorigenesis of cervical cancer (CC) [197]. Induced TXNIP expression suppresses cell proliferation, migration and invasion [198].
Antitumor	Lung cancer	Decreased in cancer tissues by DNA methylation and histone modification	TXNIP expression is associated with clinical stage in lung cancer [199], and upregulation of TXNIP induces cell cycle arrest and inhibits cell growth and metastasis [200, 201]. Suggested mechanism is that TXNIP promotes A2BR degradation and inhibits cRaf /Erk signaling [202].
Antitumor	Esophageal cancer	Higher expression levels in cases treated with neoadjuvant chemotherapy compared to untreated.	TXNIP expression is negatively correlated with lymph node involvement and perineural invasion in tumors receiving primary surgery only and positively associated with favorable disease-specific survival in chemotherapy-treated cases [203]. Induction of TXNIP expression prevents tumorigenesis possibly by promoting DNA damage and apoptosis [204, 205].
Antitumor	Osteosarcoma	Downregulated in osteosarcoma	Lower TXNIP expression is associated with poor prognosis [206]. TXNIP overexpression inhibits cell growth and migration by repressing the Warburg effect [120] and promotes drug sensitivity by inducing senescence [207].
Antitumor	Head and neck cancer	Decreased in head and neck squamous carcinoma (HNSCC)	TXNIP is highly methylated in HNSCC samples [208], and induced TXNIP expression enhances radiotherapy [209].
Anti-tumor	Kidney cancer	Downregulated in renal cell carcinoma (RCC)	TXNIP expression positively correlates with improved overall survival (OS) and disease-free survival (DFS) [210] and increases apoptosis [12].
Antitumor	HCC	Repressed by HDACs in HCC	TXNIP mediates acetylation inhibitor-induced suppression of HCC by triggering potassium deprivation [211] and suppression of cancer stemness [87] and aerobic glycolysis [212].
Antitumor	Melanoma	Decreased expression in melanoma progression and metastasis	TXNIP limits invasive potential and metastasis of melanoma cells by modulating metabolic state and redox homeostasis [160, 213], and enhances drug sensitivity [176].
Antitumor	Gastric cancer	Higher incidence of gastric cancer in *Txnip*-KO than in wild-type mice	TXNIP supresses gastric carcinogenesis by decreasing the production of pro-tumor inflammatory mediators (TNFα, NF-κB and COX-2) [32], and inhibiting proliferation and invasion by increasing ROS levels [214].
Antitumor	Pancreatic adenocarcinoma	Decreased in pancreatic adenocarcinoma (PDAC) distant metastases	TXNIP suppresses tumor progression and metastasis by inhibiting glucose metabolism [89, 123].
Antitumor	Colorectal cancer	Decreased expression in colorectal cancer (CRC)	TXNIP inhibits cell migration and invasion by decreasing Trx-1 expression and nuclear localisation [215], and promotes the differentiation of cancer cells by inhibiting glycolysis [85].
Pro-tumor	AML	Increased expression in virus-induced murine model, and in certain human subtypes.	TXNIP upregulation contributes to the development of virus-induced murine leukemia and certain subtypes of human AML mainly characterised by t (8; 21) [216].
Pro-tumor	Bladder cancer	Increased expression and can be induced by arsenite treatment	Arsenite treatment causes the upregulation of TXNIP and subsequent activation of NLRP3 inflammasome, which accounts for an increased expression of EGF, TGFα, and HSP90 [217].
Pro-tumor	Lung cancer	Upregulated in non-small cell lung carcinoma (NSCLC) cell lines under hypoxic conditions	TXNIP expression is significantly positively correlated with HIF-1α expression, with high expression associated with decreased shorter PFS [20].
Pro-tumor	Kidney cancer	74% cases without expression, remainder displaying medium or strong expression	TXNIP expression is positively correlated with shorter DFS in conventional RCC [22].
Pro-tumor	HCC	Increased expression in HCC cases, and mesenchymal-like highly motile and invasive HCC cell lines	The overexpression of TXNIP promotes migration by upregulating ROS levels [21].
Pro-tumor	Gastric cancer	Increased expression	TXNIP expression is negatively associated with clinical outcome, especially for stages 2-4 [14].
Pro-tumor	Pancreatic adenocarcinoma	Upregulated in acid-adapted cancer cells	TXNIP is involved in metabolic reprogramming (towards oxidative phosphorylation) to assist cancer cell survival in acidic tumor niches [218].

In contrast, other reports show that high TXNIP levels can also correlate with poor clinical prognosis in some cancers. For example, lung cancer patients with high levels of TXNIP exhibit decreased progression-free survival compared to counterparts with low TXNIP levels (18.0 vs. 23.0 months) [20]. To be noted, just 70 samples were collected for analysis. Thus, more samples need to be used for further investigation. In HCC and conventional (clear cell) renal cell carcinoma, TXNIP overexpression promotes angiogenesis and metastasis [21, 22]. Similarly, even though high *TXNIP* expression is associated with favorable prognosis in breast, liver, and lung cancers, it correlates with poor prognosis in gastric cancer in a pan-cancer analysis. These data indicate that the roles of TXNIP in cancers show tumor specificity [14]. In addition to tumor-specific functions, TXNIP may also exert opposite functions at different stages during cancer progression. When analysing early *vs*. late-stage cases separately in ovarian cancer, TXNIP expression is associated with different clinical outcomes, namely, improved survival in early-stage disease but poor survival in late-stage disease [23].

In this review, our focus is less on deciphering TXNIP’s prognostic impact but more on the role of TXNIP within the tumor microenvironment (TME), including both tumor cells and host cells, and its impact on different cancer hallmarks. Led by the literature, we pay particular attention to the roles of TXNIP in redox, metabolic and immunological control of tumor biology. Papers that we consider to be key in understanding the role of TXNIP in cancer biology are highlighted in *italic* throughout the review.

## Regulation of TXNIP

The expression of TXNIP is regulated by a variety of biological processes and associated pathways/factors. These pathways include common regulatory mechanisms (such as transcriptional factors, microRNAs and circular RNAs, epigenetic regulators and regulators of mRNA and protein stability), oncogenes and TSGs, ER stress signaling, cytokines and growth factors. Notably, many of these regulatory signaling pathways are bi-directional. Indeed TXNIP has been reported to regulate the expression of >10 factors that regulate TXNIP’s own expression: microRNAs (miRNA-204 and miR-124a) [24–26], tumor suppressors (p53 [17, 27] and PTEN [28]), ER components, (protein disulfide isomerases [29] or apoptosis signal-regulating kinase 1 [30]), cytokines (IL-1β, IL-18 [31], TNFα and COX-2 [32]). These and other mono-directional regulators of TXNIP expression are summarised in Table 2.

**Table 2:** TXNIP regulatory signaling pathways.

Signaling pathways	Classification	Regulators
Common regulatory pathway	Transcriptional factors	MondoA[36], ChREBP [37], MLX [33], FoxO1 [37, 219], Max [220], KLF6 [221], STAT3 [39], NRF2 [222], NFATC2 [212], PAX5 [118], LKZF1 [118]
	microRNAs and circular RNAs	miR-21a [40], miR-148a [41], miR-135b-5p [42], miR-152-5p [43], miR-204 [24], miR-211 [223], miR-224 [224], miR-373 [225], miR-411-5p [226], miR-17 [55], miR-452 [213], miR-20a [213], miR-128-3p [132], miR-27a-3p [87], miR-424-5p [227], CircECE1 [120], circDCUN1D4 [228]
	epigenetic regulators	EZH2 [44], UHRF1 [12]
	regulators of mRNA and protein stability	LncRNA Gm15441 [45], LncRNA SNHG15 [46]
Oncogenes and TSGs	Oncogene	C-MYC [48], K-RAS [49], Ras [50], HER2 [51]
	Tumor suppressor	P53 [27], PTEN [28]
ER stress signaling	IRE-1a branch	IRE-1a [31, 54], XBP1 [56]
	PERK branch	PERK [57], ATF4 [229], CHOP [230]
Cytokines and Growth factors	Cytokines	TNFα [59], IL-1β [61], TGFβ1 [62]
	Growth factors	IGF1 [60]
Other regulatory conditions	Hypoxia condition	HIF-1a [65]
	mitochondrial labile iron dysfunction	CISD2 [68]
	Drug treatment	All-trans retinoic acid [69]

TXNIP expression has been strongly associated with glucose-sensing transcriptional complexes, especially the ChREBP/MondoA:Mlx complex [33]. As a result, MondoA/TXNIP signaling has been linked to the regulation of cellular glucose [34]. The factors involved in TXNIP regulation (Table 2) constitute a comprehensive regulatory network that can be broadly divided into four classes [35]: 1) transcription factors (MondoA [36], ChREBP [37], PTEN [38], MLX [33], FoxO1 [37], STAT3 [39]); 2) microRNAs and circular RNAs (miR-21 [40], miR-148a [41], miR-135b-5p [42], miR-152-5p [43], miR-204 [24]); 3) epigenetic regulators (EZH2 [44], UHRF1 [12]) and 4) regulators of mRNA and protein stability (LncRNA Gm15441 [45], LncRNA SNHG15 [46]).

### Oncogenes

TXNIP expression can also be suppressed by oncogenes [47]. For instance, in breast cancer, c-Myc has been exhibited to antagonise TXNIP expression in MondoA-dependent pathway [48]. By binding to TXNIP promoter in E-box -containing region, c-Myc competes with MondoA and represses TXNIP expression in TNBC, indeed a c-Myc^high^/TXNIP^low^ signature correlates with poor OS specifically in this subclass of breast cancer [36]. When compared with iAP mice (mice harbouring conditional null alleles of *Apc* and *Trp53*), iKAP mice (engineered with a doxycycline-inducible oncogenic *Kras* allele and conditional null alleles of *Apc* and *Trp53*) exhibit reduced TXNIP expression, suggesting that oncogenic KRAS is capable of TXNIP regulation [49]. Laio et al. go on to demonstrate that KRAS inhibits interferon regulatory factor 2 which in turn inhibits CXCL3 expression and the recruitment of CXCR2+ myeloid cells, an axis that can be used to stratify patients for efficacious anti-PD1 therapy [50]. The oncogenic GTPase Ras has also been shown to inhibit TXNIP expression by suppressing the translation of *TXNIP* mRNA [50]. Additionally, in a study of 788 node-negative patients (which showed that TXNIP expression is associated with better prognosis [HR 0.642; *p* < 0.001]), oncogenic activation of HER2 is associated with decreased TXNIP expression and a concomitant increase in reactive oxygen species (ROS) production in breast cancer [51].

### ER stress signaling

**Fig. 1:**
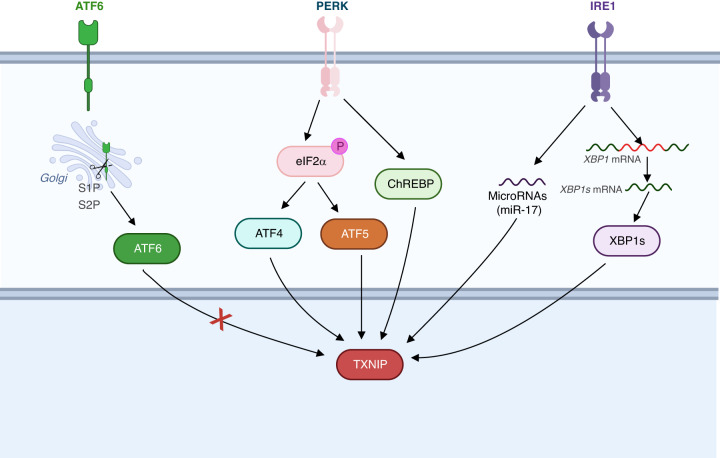
ER stress-mediated TXNIP regulation mainly depends on PERK and/or IRE-1a signaling pathways. Both PERK and IRE-1 are required for TXNIP induction in ER-stress-induced β-cell death [31]. Notably, PERK on its own can also regulate TXNIP [57]. IRE1α and its downstream effector XBP1 are also shown to be responsible for TXNIP-induced mitochondrial dysfunction, without involvement of PERK signaling [56]. Recently, IRE1α-microRNA signaling axis (miR-17) has been described to control TXNIP expression [55].

ER stress signaling is regulated by three major functional sensors: activating transcription factor 6 (ATF6), inositol-requiring enzyme 1α (IRE1α) and protein kinase R-like ER kinase (PERK) [52]. Under homeostatic conditions, the luminal ER master chaperone protein Binding immunoglobulin Protein (BiP) is bound to these sensors, maintaining sensors in an inactive state. Under ER stress conditions, misfolded proteins accumulate in the ER lumen and bind with high affinity to BiP, resulting in displacement of BiP and the activation of ER sensors. This ultimately leads to transcriptional reprogramming to maintain ER homeostasis, a process known as the unfolded protein response (UPR) [52]. The UPR is an evolutionarily conserved cell stress response, but aberration in the activation of ER stress is a key driver of tumorigenesis and reprogramming of the TME [53]. TXNIP signaling is implicated in ER stress, participating in the different branches of the UPR. Both PERK and IRE-1 are required for TXNIP induction in ER-stress-induced β-cell death [31], while activation of ATF6 signaling fails to induce TXNIP expression. Importantly, PERK and IRE-1α are able to regulate the expression of TXNIP via eIF2α-ATF5 and XBP1 signaling pathways, respectively [54–57] (Fig. 1).

### Cytokines and growth factors

Cytokines play a crucial part in immunity and the TME by mediating cell-to-cell communication [58]. The signaling driven by inflammatory, regenerative, and anti-inflammatory cytokines modulate the recruitment, development, and behavior of different cell types from the innate and adaptive immune pools. TXNIP expression is regulated by cytokines to achieve various functions. In naïve T cells, TNFα triggers TXNIP downregulation leading to increased glucose uptake and further T cell stimulation [59]. Insulin-like growth factor 1, a growth factor known to promote cancer development, negatively regulates TXNIP expression enhancing its antiapoptotic effects [60]. In addition, IL-1β and TGFβ1 suppress TXNIP activation in fibroblasts and mesenchymal progenitors, respectively [61, 62]. However, TGF-β1 can also induce TXNIP expression to achieve transcriptional repression in HL-60 cells [63].

### Other regulatory conditions

Additional endogenous and environmental factors have been reported to induce TXNIP expression. In energy-depleted conditions, AMP-activated protein kinase induces the degradation of TXNIP [64], while under hypoxic conditions [65, 66], HIF-1α induction has been shown to increase TXNIP expression. Inversely, TXNIP also causes the degradation and export of HIF-1 α, suggestive of another bi-directional regulatory loop [67]. The CISD2 (NAF-1, nutrient-deprivation autophagy factor-1) protein is reported to regulate TXNIP expression through a process that involves the perturbation of mitochondrial labile iron, mitochondrial ROS and triggered ferroptosis in breast cancer cells [68]. Retinoic acid-mediated TXNIP suppression is found to de-activate hepatic stellate cells and thereby help prevent liver fibrosis and carcinogenesis [69].

**Fig. 2:**
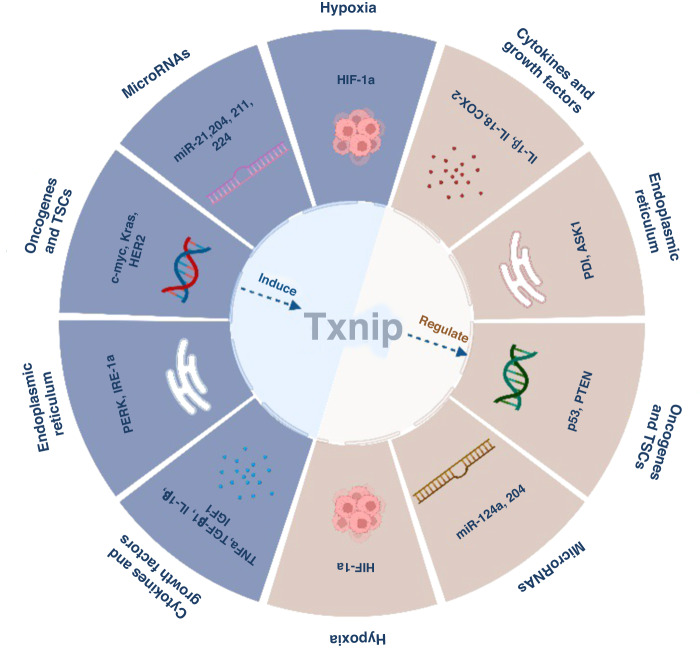
TXNIP is regulated by diverse factors and the regulation is bi-directional. TXNIP expression is regulated by a variety of different signaling pathways, including microRNAs, oncogenes and TSGs, cytokines and growth factors, endoplasmic reticulum and some specific environmental conditions (e.g. hypoxia). Additionally, TXNIP also regulates these pathways as part of a feedback loop to attenuate or amplify signaling. For example, oncogenes, including Kras, HER2 and c-Myc, induce TXNIP expression, while TXNIP can regulate the expression of p53 and PTEN. Moreover, HIF-1a and TXNIP can regulate each other under different conditions.

In conclusion, TXNIP expression and, therefore, function is regulated by diverse factors associated with different tissues and conditions (Fig. 2), and a complex network of positive and negative regulatory loops.

## Biological roles of TXNIP

**Fig. 3:**
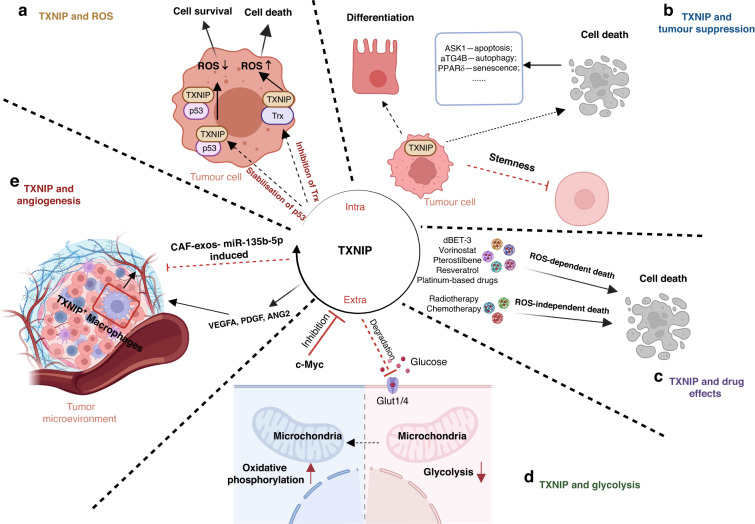
TXNIP is closely involved in various biological processes. **a** TXNIP can positively or negatively regulate oxidative stress via binding with either Trx or p53; **b** the activation of TXNIP leads to tumor suppression by affecting cell differentiation, cell stemness and cell death (such as apoptosis, autophagy, and senescence); **c** TXNIP mediates drug-induced cell death via ROS-dependent/- independent pathways; **d** TXNIP impacts on cellular metabolism, transforming cells from glycolytic to reliant on oxidative phosphorylation, by regulating the expression of GLUT1/4; **e** TXNIP increases the expression of VEGFA, PDGF, and ANG2.

TXNIP has been seen to be involved in a myriad of cellular responses, including oxidative stress, differentiation, angiogenesis, apoptosis and glycolysis (Fig. 3).

### TXNIP and oxidative stress

As discussed, TXNIP was originally identified as a key regulator of cellular redox and its expression has subsequently and consequently been shown to be closely associated with intracellular ROS levels [14]. *This regulation is mediated by its antagonistic effects on Trx by an intermolecular disulfide interaction, meaning TXNIP-Trx binding increases the presence of ROS* [44, 70]. A study, which assessed blood samples from chronic lymphocytic leukemia patients, demonstrated that TXNIP levels robustly correlated with ROS production [71]. Moreover, silencing of TXNIP has been demonstrated to decrease ROS levels in macrophages [72], but overexpressed TXNIP causes high oxidative stress, leading to DNA damage, cell death [73], and autophagy-related apoptosis [74]. However, TXNIP has also been shown to bind and stabilise p53 protein, showing antioxidant effects and further maintaining the cell survival of the hematopoietic cells [75]. Taken together, TXNIP can either promote or inhibit the production of ROS by binding to either Trx or p53, leading to cell death or cell survival, respectively. Notably, the dual role of ROS in cancer could partially be the reason for diverse functions of TXNIP in cancer [76].

### TXNIP and tumor suppression

TXNIP can induce cell death and inhibit proliferation, thus being regarded as a TSG. TXNIP activation leads to G1/S phase arrest by modulating cell cycle regulatory proteins (such as p27kip1, JAB1, CDK2, and cyclinE) [77]. In contrast, loss of TXNIP facilitates rapid cell division and activation of DNA replication, leading to cell proliferation in breast and lung cancer models [78, 79]. *After shuttling into the mitochondria, TXNIP binds to thioredoxin and abolishes its inhibitory effect on ASK1-mediated apoptosis* [30]. In addition, TXNIP is also involved in autophagy and senescence [80–82]. Mechanistically, TXNIP interacts with REDD1 to promote mitochondrial rearrangement and ROS production, suppressing ATG4B catalytic activity and inducing autophagy [83]. Moreover, TXNIP can promote the differentiation of leukemia-initiating cells and CRC cells in glycolysis-independent and glycolysis-dependent manners, respectively. TXNIP-dependent cell differentiation in leukemia and CRC promotes the suppression of leukemogenesis and reduces CRC cell viability [84–86]. Additionally, a reduction in TXNIP induced by M2 macrophage-derived exosomes has been observed to be critical for maintaining cancer “stemness” and promoting tumor progression in HCC [87].

TXNIP has also been reported to reduce the migratory capacity of tumor cells. *Downregulation of TXNIP maintains the Trx/Trx reductase (Trx/TrxR) system in an active state, driving epithelial-mesenchymal transition and increasing the metastatic potential of cancer cells* [88]. In pancreatic cancer, elevated TXNIP expression leads to repression of malignant transcripts and impairment of metastatic tumorigenesis through the epigenetic reprogramming of chromatin [89]. Similarly, albeit through a different mechanism, TXNIP mediates the internalisation and degradation of EGFR, decreasing the migratory capacity of breast cancer cells [90]. Interestingly, breast cancer cell-derived exosomes negatively regulate TXNIP expression, resulting in the activation of the WNT/β-catenin pathway in fibroblasts and induction of cancer-associated fibroblasts (CAFs) [91]. These CAFs then promote cancer cell invasion and metastasis [91]. However, another study, this time in HCC, observes that TXNIP expression is positively associated with the migratory and invasive ability of hepatocellular cancer cells [92], stressing the importance of underlying tissue and cell type in determining the impact of TXNIP function on migration.

TXNIP can also affect tumorigenesis through its association with metabolic disorders. Epidemiological and clinical studies highlight that cancer patients with diabetes have a higher morbidity and mortality [93]. The mechanisms of diabetic stress-associated tumor progression and metastasis include inhibition of antitumor immune responses [94, 95], metabolic transcriptional modulation of cancer cells [96], decellularization of extracellular matrix scaffolds [97], and even vascular dysfunction [98]. The master roles of TXNIP in fasting, insulin sensitivity, and β-cell apoptosis are well known, and these functions have been linked to an increased risk of diabetes and other metabolic disorders [99–102]. These data collectively suggest TXNIP acts as a driver of metabolic diseases, contributing to the development of cancers [35, 103].

### TXNIP and chemotherapy

Interestingly, cancer cells, displaying high baseline levels of ROS, are vulnerable to further damage caused by ROS accumulation. In this vein, a number of studies have shown that increased TXNIP expression can enhance the cytotoxicity of chemotherapeutic reagents by manipulating ROS levels, as the levels of ROS in cancer cells provide a potential therapeutic vulnerability. This antitumor strategy has already been exploited by several agents, including dBET-3, vorinostat, pterostilbene, and resveratrol [104–107]. Additionally, platinum-based drugs can also inhibit the activity of TrxR; a process that has been demonstrated to be critical in promoting antitumor effects [108–110].

TXNIP can also promote treatment efficacy in a ROS-independent fashion. In esophageal cancer, cisplatin treatment leads to TXNIP upregulation, mediating its cytotoxicity via an unknown mechanism [111]. In oral cancer models, overexpression of TXNIP enhances the effectiveness of radiotherapy via the DNA repair pathway [112]. Compared to cisplatin-sensitive cells, cisplatin-resistant cells exhibit downregulation of *TXNIP* mRNA mediated by UCA1, suggesting a role of UCA1 and TXNIP in contributing to cisplatin resistance in lung adenocarcinoma [113]. In support of these findings, exogeneous overexpression of TXNIP in glioma cells decreases the median inhibitory concentration (IC50) of cisplatin [114]. Combining a TXNIP agonist, D-Allose, with chemotherapy or radiotherapy results in enhanced antitumor effects in both head and neck and lung cancer models [115, 116]. These studies collectively suggest that increased TXNIP expression mediates or enhances the cytotoxicity of chemo-radio therapies.

### TXNIP and glycolysis

Metabolic reprogramming is a hallmark of cancer development and metastasis and TXNIP-dependent metabolic phenotypes are associated with patient prognosis. Elevated glycolysis is closely associated with the initiation of cancer, producing glucose-dependent adenosine triphosphate (ATP) and glycolytic intermediates for macromolecular biosynthesis. c-Myc, a well-known modulator of metabolism, mediates metabolic and phenotypic changes in cancer [117]. TXNIP is reported to both regulate lipid and glucose metabolism directly [118, 119] and mediate c-Myc-driven metabolic changes indirectly [20, 120–122]. For instance, a study in TNBC identified that TXNIP suppression by MYC can reprogramme the metabolic phenotype of cancer cells [36]. Additionally, in ER^+^ breast cancer, the levels of TXNIP expression in tumor cells are associated with different metabolic subtypes [54]. In MCF7 cells, which have high basal TXNIP expression, an elevated mitochondrial oxidative phosphorylation (OXPHOS) phenotype is observed. In contrast, T47D cells, which have low expression of TXNIP, display an aerobic glycolysis phenotype [54]. Interestingly, estrogen has been shown to repress TXNIP expression and drive the Warburg effect [54]. In pancreatic cancer, the tumor suppressor FBW7 (F-box and WD Repeat Domain-Containing 7) exerts its antitumor effects by controlling glucose metabolism and oxygen consumption in a TXNIP-dependent manner [123]. More importantly, it should be mentioned that genetic deletion of TXNIP increases the uptake of glucose by regulating the expression of HIF-1a or c-Myc, which leads to the metabolic reprogramming towards aerobic glycolysis [119, 124, 125]. Collectively, these data highlight the dominant role of TXNIP in controlling glucose homeostasis [48].

Further understanding reveals one of the molecular mechanisms is the association between TXNIP and GLUT family. The GLUT membrane transporter family is crucial in facilitating glucose transportation and includes class I (GLUT1-4), class II (GLUT7, GLUT11) and class III (GLUT6, GLUT8, GLUT12) transporters [126]. TXNIP inhibits the influx of glucose and lactate by decreasing the expression of class I glucose transporters like GLUT1 and GLUT4 via both endocytosis and degradation of protein levels and reduction of mRNA levels [64, 127, 128]. Recently, a class III transporter, GLUT8, a central regulator of metabolism, has also been identified to interact with TXNIP to enable hexosamine homeostasis [129]. Extracellular matrix remodelling is another critical factor governing extrinsic metabolic regulation. Defects in matrix attachment affect cellular metabolism, resulting in a reduction in glucose uptake and subsequent ATP deficiencies [130]. Matrix digestion reportedly destabilises TXNIP and enriches GLUT1 transporter at the plasma membrane to promote glycolysis; a process which is fundamental for both embryogenesis and tumorigenesis [130, 131]. All these observations emphasise the critical role of TXNIP in metabolic reprograming.

### TXNIP and tumor angiogenesis

Angiogenesis, another hallmark of cancer, enables tumors to meet nutrient and oxygen needs to sustain proliferative and metabolic requirements. In conventional RCC, immunohistochemical staining of 691 patient samples revealed that patients with high TXNIP expression have a marked reduction in tumor free survival and a higher occurrence of metastasis. Interestingly, this study showed a significantly positive correlation between TXNIP expression and inefficient vascularisation favouring tumor cell survival in RCC [22]. Notably, this study was an observational study, lacking in-vitro and in-vivo experiments. Thus, this report might not be very evident regarding the impact of TXNIP on angiogenesis. But we have to admit that it brings great significance by providing clinical support. Mechanistically, TXNIP overexpression leads to upregulation of angiogenesis-related proteins (VEGFA, PDGF and ANG2), along with an angiogenic phenotype [132]. Moreover, in osteosarcoma, single-cell RNA sequencing analysis identifies different functional subtypes in the myeloid compartment [121]. Among them, TXNIP^+^ macrophages tend to be M2-like (a broadly anti-inflammatory phenotype) and express M2 signature markers, including MERTK, MRC1, STAB1 and CD163. Furthermore, ligand-receptor interaction analysis identifies an association between TXNIP^+^ macrophages and angiogenic endothelial cells, suggesting TXNIP^+^ macrophages may facilitate angiogenesis [121]. However, exogenous TXNIP expression in CRC lines (LoVo and HT29) represses angiogenesis [42]. Similarly, inhibition of a cyclin-dependent kinase transcriptionally represses TXNIP expression and promotes endothelial cell invasion, migration and vascular sprouting in breast, lung and prostate cancer cell lines [122]. Thus, TXNIP’s role in regulating angiogenesis is context dependent.

## Immune regulation by TXNIP

**Fig. 4:**
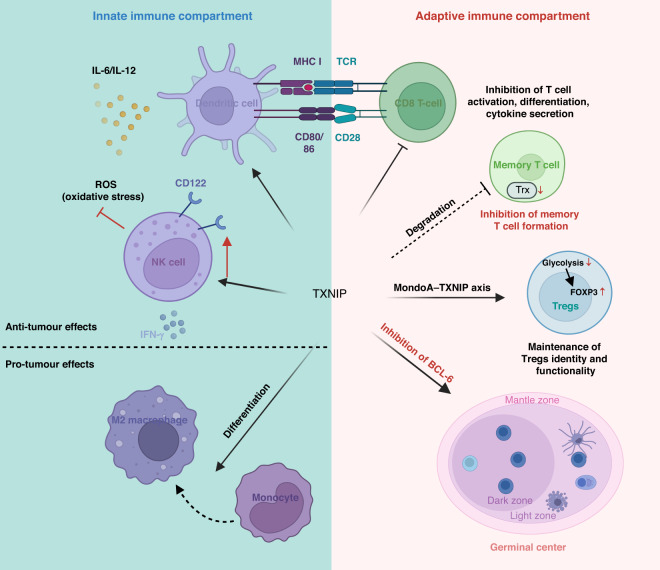
TXNIP plays important roles in both innate and adaptive immune regulations. Schematic summarising the impacts of TXNIP on different arms of the immune system. TXNIP can maintain the survival and promote the activation of NK and dendritic cells (DC), leading to increase cytotoxicity [148, 151]. Meanwhile, TXNIP facilitates the differentiation of monocytes to M2 macrophages, creating a pro-tumoral microenvironment [154]. Moreover, TXNIP is involved in the development of various T and B cell subsets. It is essential in maintaining the identity of Tregs [163], while inhibiting the formation and activation of memory T cells and CD8^+^ T cells [156, 159]. Through inhibition of BCL-6, TXNIP can promote the formation of the germinal center [168].

An increasing number of studies are unveiling the impact of TXNIP expression on the immune system. A pan-cancer study recently reported a correlation between TXNIP and infiltration of immune cells, supporting the idea that TXNIP may be an important player in determining the immunological makeup of the TME [14]. In addition to its regulation of immune-related signaling pathways and cytokine production, TXNIP is also demonstrably involved in the development and maturation of innate and adaptive immune cells (Fig. 4). By impacting different immune cell in different ways, TXNIP can drive both antitumor and pro-tumor effects.

### TXNIP, NF-κB, and NLRP3 inflammasome signaling

TXNIP can exert effects on the immune system in several ways. *As an intracellular amplifier of oxidative stress and inflammasome activation* [133]*, TXNIP is detected in different cell types (such as tumor cells, immune cells and stromal cells)*. In endothelial cells, for example, nuclear translocation of TXNIP leads to NF-κB activation, which facilitates the expression of pro-inflammatory cytokines such as IL-1β [134, 135]. However in tumor cells, TXNIP suppresses TNF-α-induced NF-κB activity and subsequently inhibits hepatocarcinogenesis [79, 136].

Activation of the NOD-like receptor (NLR) family pyrin domain containing 3 (NLRP3) inflammasome is observed under diverse physiological and pathological conditions, such as caloric restriction [45], type 2 diabetes [137], preeclampsia [138], Alzheimer’s disease [139] and cancer [140]. It has been established that the NLRP3 inflammasome is involved in many cancer-immune relationships [141], with both antitumorigenic and pro-tumorigenic roles. On one hand, NLRP3 contributes to various types of cell death, like pyroptosis, apoptosis, necroptosis, and ferroptosis [142]; on the other, although inflammasome-inducing IL-1β can activate DCs to facilitate adaptive antitumor immune activation [143], it also expands myeloid-derived suppressor cells (MDSCs) [144].

*Numerous studies have uncovered a link between TXNIP and NLRP3 inflammasome activation, mostly due to the functions of the Trx1/TXNIP axis in ROS regulation* [100, 145]. However, this is not always the case, as Trx1 can lead to NLRP3 inflammasome activation independently of TXNIP [146]. STING triggers the TXNIP-NLRP3 interaction, leading to inflammasome activation without the involvement of Trx1 [2]. Similarly, CXCR4 can also directly bind to TXNIP and induce NLRP3 inflammasome activation without affecting the activity of Trx1 [147]. UPR signaling is another system that regulates inflammasome activation via TXNIP-dependent mitochondrial dysfunction, rather than through direct modulation of ROS levels [56]. Together, these findings indicate both Trx1 and TXNIP can also induce the activation of the NLRP3 inflammasome independently of the Trx1/TXNIP relationship and ROS regulation.

### TXNIP and innate immunity

In addition to its roles in NF-kB and inflammasome-mediated inflammation, TXNIP is also involved in regulating the generation, development and functionality of innate immune cells. *Txnip*^−/−^ mice carry a severely reduced number of NK cells [140]. There was also a decreased expression of IL2RB (CD122), but, intriguingly, the development of T and B cell populations was not impaired [148]. The reduced number of *Txnip*^−/−^ NK cells observed in this study were also shown to be functionally impaired when considering cytotoxicity and IFN-γ production [148]. Another study has also demonstrated a similar requirement of TXNIP in the effective differentiation of NK cells [149]. *Moreover, given that the core of tumors contains high levels of ROS that are associated with the presence of NK cells, TXNIP has been suggested to be an important factor governing the infiltration of NK cells into the TME* [150]. The mechanism by which tumor-infiltrating NK cells gain resistance to oxidative stress is through retention of nuclear TXNIP leading to higher Trx-1 activity [150].

TXNIP is also reported to regulate the development of myeloid lineage. A study using *Txnip*-deficient mice demonstrated the requirement for TXNIP in maintaining normal functions of DCs, including secretion of the cytokines IL-12 and IL-6 and subsequent activation of T cells [151]. When comparing gene signatures between non-activated polymorphonuclear myeloid-derived suppressor cells (PMN-MDSCs) and activated ones from the same murine models, TXNIP expression is a significantly upregulated differential in the activated group. *The authors felt these findings may tentatively indicate that TXNIP may have a role in maintaining immune-suppressive activity* [152]. Tumor-associated macrophages (TAMs) are abundant in the TME of solid tumors and promote tumor development by suppressing immune responses and facilitating tumor growth and metastasis [153]. In PDAC, TXNIP expression is upregulated in TAMs, and this is driven by *KRAS* activity in cancer cells [154]. The study goes on to demonstrate that the high expression of TXNIP in TAMs contributes to metabolic changes which are required for macrophage polarisation and the promotion of pro-tumor responses [154]. Collectively, these studies suggest a requirement for TXNIP/txnip in NK cell development and function, along with TXNIP-mediated promotion of suppressive myeloid phenotypes.

### TXNIP and adaptive immunity

The role of TXNIP in adaptive immunity appears to be more complex than in the innate compartment. In melanoma, TXNIP expression is enriched in memory T cells [155]. This may be due to TXNIP’s role in regulating CPT1a’s metabolic functions which are essential for protective memory T cell generation [156, 157], however, *TXNIP has been observed to inhibit CPT1a expression, resulting in inhibited generation of memory T cells* [157]. Dual anti-CD3/anti-CD28 stimulation on T cells suppresses TXNIP expression, and this has been attributed mainly to anti-CD3, suggesting anti-CD28 co-stimulation has minor effects [157, 158]. The activation of T cells may, at least to some extent, involve anti-CD3-mediated suppression of TXNIP, which potentially abolishes inhibitory impacts of TXNIP on transcriptional activation of targeted genes involved with T cell activation, differentiation, cytokine signaling as well as cell death pathways [159]. Notably, despite T cells showing higher levels of glucose uptake with anti-CD3/anti-CD28 stimulation, these metabolic changes are independent of TXNIP-mediated regulation of glycolysis [158].

Co-stimulatory signals are required for full TXNIP-dependent activation of T cells after TCR-MHC complex engagement, including signals from the tumor necrosis factor receptor superfamily (TNFRSF) members [157]. In a similar manner, TLR2, 4, and 5 agonists partially inhibit TXNIP expression through TNFα production [59]. The potential mechanism is likely to involve the downregulation of TXNIP, cell cycle entry and metabolic changes which are optimal for T cell proliferation and activation [59]. *TXNIP also appears to be indispensable in the restriction of T cell (mainly in CD4*^*+*^ *T cells) and germinal center B cell expansion following viral infections, a process that relies on Trx1/TXNIP balance* [158]. Additionally, similarly to the study reported by Yang et al. [150], this study also observed that ablation of TXNIP does not affect the development and homeostatic maintenance of T cells, B cells and myeloid cells [158]. Importantly, with regards checkpoint therapy, the levels of TXNIP have recently been reported to be negatively associated with the expression of PD-L1, indicating the potential impacts of TXNIP on immunomodulatory proteins [160]. However, whether or not other immune checkpoints are regulated by TXNIP needs further elucidation.

**Fig. 5:**
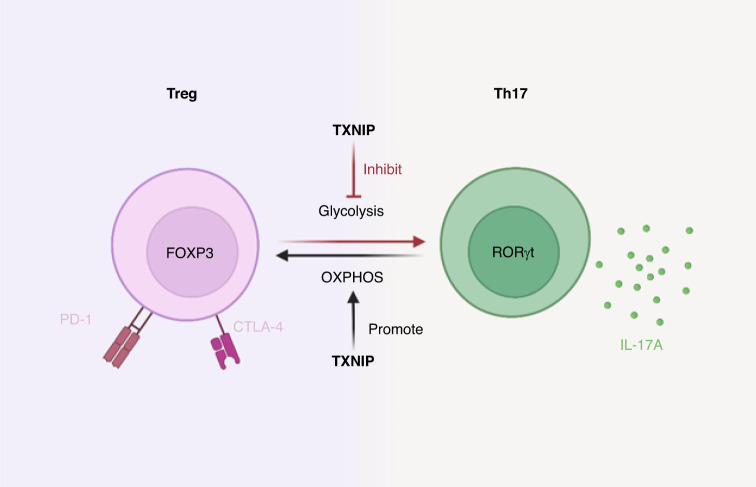
Cellular metabolism contributes to the plasticity of Tregs. The expression of TXNIP in Tregs determines their metabolic state. Low expression of TXNIP in Tregs promotes glycolysis, facilitating Th17 inflammation, whilst high TXNIP expression in Tregs switches the cells towards OXPHOS, helping to maintain suppressive function.

Regulatory T cells (Treg) are immunosuppressive cells which regulate multiple arms of the immune system with a particular emphasis on cytotoxic T cell responses. The impact of Tregs in tumorigenesis varies depending on the specific subtype of Tregs; consequently they are predictive of a variety clinical outcomes with an overall strong trend towards the prognostically negative [161, 162]. The plasticity and stability of Tregs are regulated, at least in part, by cellular metabolism [162]. *A recent study highlights the requirement of the MondoA-TXNIP axis in maintaining the identity and functionality of Tregs by repressing glycolysis in CRC* [163]. Inhibition of MondoA or TXNIP in Tregs leads to the upregulation of glycolytic genes and the increase of glycolytic activities, which compromises immuno-suppressive functions in these cells [163]. Fascinatingly, glycolysis reduces FOXP3 and RORγτ expression in Tregs, promoting a switch to a Th17-like effector phenotype, which can be reversed by TXNIP activation [163]. Accordingly, intra-tumoral Tregs generally present with increased induction of glycolytic pathways, resulting in a pro-tumor immune microenvironment [163, 164] (Fig. 5).

Germinal centers (GCs) are the main sites of antigen-stimulated B cell proliferation and differentiation. In GCs, antigen-activated B cells not only produce high-affinity antibodies through somatic hypermutation (SHM) on immunoglobulin genes, but also produce antibodies with specialised functions via class-switch recombination (CSR). GC B cells express high levels of BCL-6 which modulates GC formation through several different mechanisms, including inducing the GC to undergo SHM and CSR, supressing premature B cell activation prior to GC formation and inhibiting B cell differentiation [165–167]. *TXNIP is reported to promote GC development by suppressing BCL-6 activity* [168]. *Txnip*^−/−^ mice are reported to exhibit large secondary follicles with a GC-like structure and a higher population of Ki-67^+^ B cells in the spleen [168]. TXNIP has also been shown to be expressed at different stages of B cell development [118]. As a central metabolic gatekeeper, TXNIP restricts glucose and energy supply, which are essential for pre-B cell development [118]. Additionally, deletion of TXNIP provides strong survival advantage and rescues prednisolone-induced cell death in pre-B Acute Lymphoblastic Leukemia (ALL) cells due to removal of ATP production [118]. Collectively, TXNIP is involved in the maintenance and activation of different adaptive immune cell types, with its main impact potentially being on metabolic and subsequent phenotypic changes.

## TXNIP-targeting therapeutics

As we have discussed, it is clear that TXNIP is associated with multiple biological functions that are critical for the development of several pathological processes. Consequently, there are a number of therapeutic strategies currently aimed at modulating TXNIP expression/functions for clinical application.

TXNIP can contribute to disease by regulating oxidative and glycolytic stress, inflammation, and by inhibiting the cell cycle. These notions are supported by cumulative evidence that loss of TXNIP by pharmacological inhibition or genetic perturbations results in amelioration from neurological disease and diabetes in murine models [3, 169]. TXNIP antagonists have been comprehensively reviewed by Qayyum et al. [170], and consist of small-molecule drug, phytochemicals, and peptides. Two well-characterised drugs, verapamil (NCT02372253) and Taurine (NCT01226537), that modulate TXNIP levels are currently being tested in clinical trials for the treatment of type 1 and 2 diabetes. Verapamil, a non-dihydropyridine L-type calcium channel blocker traditionally used orally for the treatment of hypertension, inhibits TXNIP expression [171]. A recent high-throughput screen has identified another TXNIP inhibitor SRI-37330, which significantly decreased TXNIP expression, glucagon secretion, and hepatic glucose output, thereby being championed as a potential treatment for diabetes [172]. Interestingly and in contrast to these inhibitors, taurine, used for glycaemic control in diabetic patients, is reported to increase TXNIP expression [173].

In the context of cancer treatment, TXNIP agonists hold great potential as antitumor agents. Vorinostat, a pan histone-deacetylase inhibitor, and rapamycin, an mTORC1 inhibitor, have been shown to limit disease progression in Ras-driven cancers, with the ability to induce TXNIP expression [105]. Targeted therapies in breast cancer such as trastuzumab, cetuximab, and lapatinib, which block the Her-1/2 pathway, can cause G1 cell cycle arrest and also highly increase TXNIP expression [174]. Additionally, in TNBC, silibinin, commonly used in the treatment of toxic liver damage, has been shown to upregulate TXNIP, which suppresses glycolysis and cell proliferation [175]. BRAFi, which has been approved to treat advanced melanoma and proved to show strong clinical benefit in BRAF^v600^ melanoma [48, 176, 177], can also induce TXNIP expression through regulating the association between MondoA and *TXNIP* promoter [176]. Notably, the expression level of TXNIP is associated with favorable clinical response to BRAFi [176]. With a renewed emphasis on therapies which modulate the tumor metabolome, these and additional TXNIP agonists may show great potential.

## Conclusion

TXNIP is a multifaceted protein involved in several fundamental biological processes and therefore is potential pharmacological target for multiple applications. Its ability to regulate glycolytic stress, oxidative stress, ER stress and the cell cycle gives it a central role in balancing different cell states, leading to various cell fates. Accordingly, TXNIP can drive both beneficial and detrimental effects in different pathologies, like metabolic diseases and cancer. Consequently, TXNIP antagonists are candidates for treating diabetes and neurological diseases, whereas TXNIP agonists hold potential for cancer treatment.

TXNIP regulatory networks are complex and feedback loops render them mutually regulatory. Apart from the mechanisms reviewed by Masutani et al. [35], oncogenic and tumor suppressor genes, ER stress signaling, cytokines, and other conditions like hypoxia also modulate TXNIP expression. Oncogene-mediated downregulation of TXNIP is mostly associated with inhibition of cell death and an increase in cellular metabolism, which favour tumor proliferative abilities and resistance to anticancer treatment [48]. Cellular recovery from ER stress via UPR signaling alters TXNIP as well. So far, two of three UPR transducers are mainly reported in TXNIP regulation, namely PERK and IRE-1a [31]. In certain conditions, they work together or separately for TXNIP alteration. Several effectors lie at the downstream signaling of PERK-eIF2a axis to determine cell fate [178]. ATF4 is a well-known early acute UPR and terminal-UPR mediator and reported to be required for TXNIP-mediated NLRP1 inflammasome activation instead of NLRP3 inflammasome activation [178, 179]. The literature reviewed here suggests that the stability of TXNIP serves as key switch between terminal UPR and adaptive UPR, with clear integrative mechanisms requiring further elucidation. Cytokines not only regulate survival, proliferation, differentiation and the function of immune cells, but also contribute to reshaping the TME [180], including through their exosomal binding [181]. Exosomes and TXNIP also have an intimate relationship as several miRNAs shuttled in exosomes mediate TXNIP downregulation, which can complement the effects of cytokines in the tumor microenvironment [42, 87, 91]. The regulation of TXNIP expression, therefore, appears to be under the control of a plethora of inter-cellular signals (multiple cytokines and exosomal miRNAs), which makes it complex to establish the exact role of TXNIP in tumor microenvironment-driven tumor progression [182].

The molecular mechanisms of TXNIP regulation of cell cycle, inflammation and glycolysis can have tremendous consequences on both tumor and immune cells. With important roles in several cancer types, TXNIP affects cell proliferation and death, drug sensitivity, angiogenesis, and glycolysis [16]. Consequently, TXNIP is closely involved in the remodelling of the TME, especially the immune compartment. The importance of immune contexture has been emphasised in cancer control in recent years [182]. Other than the interaction between TXNIP and NF-κB or inflammasome signaling, the roles of TXNIP in both innate and adaptive immune modulation suggest its potential role as a target for drug discoveries. NK cells and DCs are two specialised innate immune cell types, acting as the main effector and antigen-presenting cells, respectively [183, 184]. Deletion of TXNIP restrains the development and maturation of NK cells and functions of DCs, which causes the dysfunction of antitumor immunity [148, 151]. However, TXNIP is also observed in activated PMD-MDSCs and potentially associated with their suppressive activity [152]. As well, TXNIP inhibits the generation of protective memory T cells via degradation of CPT1a [157]. Moreover, TXNIP affects the proliferation and activation of T cells, and is crucial to maintain Tregs identity and its immune-suppressive function [163]. In addition, TXNIP is involved in GCs formation and development of B cells at different stages [118, 168].

In summary, as a central element receiving inputs from multiple extracellular signals, and acting as an intracellular hub for ROS homeostasis, metabolic responses, stress integration, immune functions, and cellular outcomes, TXNIP holds a crucial and pivotal role in health and disease. TXNIP offers an attractive point of pharmacological intervention. Future studies and clinical trials in humans will eventually translate the vast scientific research in the field of TXNIP regulation, into tangible outcomes for the benefit of multiple patient groups.

### Authors’ comment

We set out to write as comprehensive a review as possible, and although there are contradictory reports, a consensus view as to TXNIP’s role in cellular and tissue homeostasis emerges. It’s central role can be clearly seen in maintaining cellular health in a supportive tissue environment, post-stress, and although not yet robustly demonstrated, it seems reasonable to hypothesise that under normal physiological conditions, the return of TXNIP to resting levels occurs quickly in co-ordination with resolution.

Interestingly, in cancers, frequently considered diseases of chronic epithelial stress, TXNIP is more commonly seen to be decreased in expression compared to healthy tissue at clinical presentation (Table 1), with additional stressors such as chemotherapy, hypoxia, or acidification seen to increase expression back towards ‘healthy’ expression levels. Given TXNIP’s central role, its loss being a positive for cancer survival is prima face contradictory, however, the key here is perhaps TXNIP’s impact on its environment.

This current “age of the TME” is increasingly revealing that successful tumors subvert their proximal, and sometimes distal, environments, indeed there is a live debate concerning whether or not certain tumors emerge because of their local environment rather than create it. Here we see that the loss TXNIP in epithelial cells has been shown to inhibit the inflammasome, but beyond this, the loss of environmental TXNIP, something that can be driven by a shared stressor or crosstalk (via common mediators), decreases NK cell generation and myeloid (including DC) activation.

Given the importance of the innate immune system in developing and maintaining the TME, this suggests to us that low environmental TXNIP is important in allowing for tumor development. Although not in cancer, an example of this “shared movement” of TXNIP across multiple cells types in the systemic environment can be seen in type 2 diabetes where high TXNIP can be seen in the PBMCs of diabetic patients, with correlations with ER stress and a common stressor, alongside inflammasome induction [185].

When viewed in the round, it is striking that TXNIP increases inflammation and antigenicity (if one accepts the PDAC TAM paper as evidence of macrophage differentiation per se) via the epithelial and innate compartments whilst simultaneously inhibiting the adaptive arms. To us, this resembles an immunological program designed for the innate-driven clearance of stressed cells whilst limiting the likelihood of adaptive-driven autoimmunity. There is an additional clue in the GC data, in that TXNIP promotes development, suggesting to us, that TXNIP may prime the adaptive arm through, for example the enhancement of local ectopic lymphoid structures, allowing adaptive immunity to react as soon as local TXNIP levels drop, if required.

Intriguingly, when considering the adaptive arm, low TXNIP is associated with increased memory T cell (CD4 and CD8) efficacy and lower numbers of Tregs, however without effective APCs or neoantigen presentation (e.g., IFNγ dependent immunoproteosomal switch) the positive impacts of these changes are minimal. A sudden increase in TXNIP expression however, through for example chemotherapy, may alter these dynamics dramatically, allowing for innate responses. If the increase is temporary, and cyclical, as with most chemotherapeutic regimes, we hypothesise that the cycles of innate stimulation/adaptive inhibition, innate inhibition/adaptive stimulation in part through TXNIP regulation may promote tumor destruction and a return to normal tissue homeostasis.

For these authors, the next steps in trying to understand the role of TXNIP in cancer, are to understand which functions of TXNIP are important in each different biological context. For example, although crudely speaking most primary carcinomas express low levels of TXNIP, what is the key reason for this—metabolism, the inflammasome, angiogenesis, or immune crosstalk? To help in this endeavor, there are variants, for example the TXNIP-T variant, that associate with an increased propensity to develop diabetes [38]. The critical cysteines for thioredoxin binding have been identified on TXNIP [186], and recent data shows that the C247S mutation protects against myocardial infarction in mice [187] whilst also regulating adipogenesis [188]. As such, the stage is now set for the field to assess the importance of thioredoxin binding in cancer models, and in so doing begin to decipher the impact of TXNIP’s diverse roles more specifically.
